# EXPLORING THE LIVED EXPERIENCES OF BLACK INFORMAL CAREGIVERS: A SCOPING REVIEW

**DOI:** 10.1093/geroni/igad104.1139

**Published:** 2023-12-21

**Authors:** Ivana Alexander

**Affiliations:** University of Maryland Baltimore, Baltimore, Maryland, United States

## Abstract

Understanding the unique caregiving experiences of Black informal caregivers, most of whom are women, and how they are influenced by the historical and sociopolitical context of the Black experience in the America is critical to building an effective care infrastructure that is inclusive. Gaps in existing literature include a paucity of studies that explore in-group differences in the caregiving experiences of Black informal caregivers as well as a dearth of qualitative studies that center their voices and lived experiences. This scoping review explored current representations of the Black informal caregiving experience in qualitative literature. It sought to address the heterogeneity in the Black caregiver experience through amplification of caregivers’ voices. Specifically, it included studies that focused on varied informal caregiving arrangements which exist for Black informal caregivers who are caring for chronically or terminally ill adults. Overall, 65 studies were selected for full-text review. After data characterization of the full-text articles, nine studies remained and were included in the analysis. Four broad themes were identified: (1) cultural beliefs, (2) religion and spirituality, (3) systemic barriers (healthcare system and care structures), and (4) caregiver health and well-being (physical and mental health). Findings underscore the need for an intersectional lens in exploring the implications of the Black informal caregiving experience. Their cultural and religious/spiritual identities are linked to the historical experience of Black people in the United States. Their caregiving experiences often involve cultural obligations and expectations that create tension between fulfilling caregiver roles and providing for their own health and well-being.

